# The Outcome of Complex Pelvic Fracture after Internal Fixation Surgery

**DOI:** 10.5704/MOJ.1603.004

**Published:** 2016-03

**Authors:** HD Ismail, MF Lubis, YP Djaja

**Affiliations:** Department Orthopaedics and Traumatology, Faculty of Medicine, Universitas Indonesia-Cipto Mangunkusumo Hospital, Jakarta

**Keywords:** Pelvic fracture, outcome unstable pelvic fracture

## Abstract

**Introduction:** Complex pelvic fracture, which has a very high mortality and even higher morbidity, needs internal fixation surgery as an integral part for its management. It was necessary to conduct a study regarding outcome of complex pelvic fractures after internal fixation surgery.

**Material & Method:** Twenty-six patients with complex pelvic fractures that had been treated with internal fixation surgery during 2011-2014 were enrolled. These patients had an open pelvic fractures or Tile type B or C pelvic fracture who had undergone internal fixation surgery with at least 6 months follow-up. Evaluation of the morbidity and functional scoring was performed using Majeed and Hannover Score.

**Results and Discussions:** Average of age was 31 years old and follow up time was 25 months. There were 7 patients with open pelvic fracture and 19 with closed fracture. Excellent Majeed Score were found on 78.6% cases in Tile B fractures and 50% in Tile C. Good Hannover Score was found in 64.3% Tile B cases and 80% Tile C cases. Fracture type was statistically insignificant with acquired sexual dysfunction (p>0.05), but significant with the chronic pain (p=0,.017). We also found that urogenital injury is associated with sexual dysfunction (p=0.005).

**Conclusions:** The outcome of complex pelvic fracture after internal fixation surgery was excellent. More than 90% patients got an excellent and good result on Majeed Score, and also very good and good result on Hannover Score.

## Introduction

Pelvic fracture is the leading cause of morbidity and mortality in musculoskeletal trauma. Among these fractures, complex pelvic fracture is a group that has a higher rate of morbidity compared to the other pelvic fracture. Complex pelvic fracture is defined as a pelvic fracture with soft tissue injury in the pelvic region, which includes urogenital structure, rectum, sigmoid, lumbosacral plexus, and retroperitoneal vessels, and accompanied with hemodynamic instability of the patient^1^. It is highly associated with chronic pain, sexual dysfunction, and infection. Its mortality rate is 33%, compared with 10-20% mortality rate for both unstable pelvic fracture and open pelvic fracture and 50% for open pelvic fracture alone^2-3^. This evidence was supported by Rothenberg *et al* who investigated 31 open pelvic fracture cases and found that the mortality rate was 42%^4^.

Treatment of complex pelvic fracture consist of bleeding management, hemodynamic restoration, stabilization of the pelvic ring, and a quick and accurate diagnosis and surgery ^5^. Fixation of the anterior and posterior segment as soon as possible yield the best result. During the early phase, external fixation is used for temporary pelvic stabilization. And when the patient’s hemodynamic state stabilized, definitive internal fixation surgery was performed, usually in 5-7 days.

There are several scoring systems that can be used to evaluate the outcome of complex pelvic fracture treatment. Among them, there are Majeed Functional score and Hannover score. Majeed Score assesses five factors, which is pain, standing, sitting, sexual activity, and working ability^6^. Hannover Score on the other hand pay attention to patient’s clinical symptoms and social reintegration. These scoring systems made it easier to evaluate the outcome from management of complex pelvic fracture, including after internal fixation surgery.

The previous studies about complex pelvic fractures mainly were focused on the survival rate. There was no study assessing the outcome of these patients especially after the definitive surgery. In this study, we assessed the outcome of open pelvic fracture and unstable pelvic fracture (Tile classification type B and type C) treated using internal fixation surgery in our center. The aim of this study was to evaluate the outcome so we can increase the quality of our treatment and also reduce the morbidity and mortality rate.

**Table I:** Subject characteristics

Characteristic	n(%)
Age Group	
• < 20	6 (23)
• 20-40	15 (58)
• >40	5 (19)
Gender	
• Male	17 (65)
• Female	9 (35)
Open or Closed Fracture	
• Open	7 (27)
• Closed	19 (73)
Tile type fracture	
• Type B	15 (58)
• Type C	11 (42)
Polytrauma	
• Yes	9 (35)
• No	17 (65)

Legend: Results after 1st paragraph

**Table II:** Comorbidities and complication

Comorbidities and complication	n(%)
Comorbidities	
• Urogenital Injury	14 (54)
• Extremity Fracture	4 (16)
• Perineal rupture	4 (16)
• Head Injury	3 (12)
• Abdomen Injury	3 (12)
Complications	
• Sexual dysfunction	10 (38)
• Chronic pain	9 (35)
• Infection	5 (19)
• Neurologic deficit	3 (12)
• Non-union	0 (0)

Legend: Results after 3rd paragraph

**Table III:** Functional Scoring

Fracture	Tile type Fracture [n(%)type B / n(%)type C]	Open or Closed Fracture [n(%)Open / n(%)Closed]
Majeed		
• Excellent	11 (78,6) / 5 (50)	4 (66,7) / 12 (66,7)
• Good	3 (21,4) / 5 (50)	2 (33,3) / 6 (33,3)
Hannover		
• Very Good	5 (35,7) / 2 (20,0)	
• Good	9 (64,3) / 8 (80,0)	

Legend: Results after 4th paragraph

**Table IV:** Association between variables

Parameter		Statistical Test	P value
Gender	Hannover score		1.0
Gender	Majeed score		0.667
Open or Closed Fracture	Majeed score		0.970
Open or Closed Fracture	Sexual Dysfunction		0.597
Open or Closed Fracture	Infection		0.082
Tile type Fracture	Sexual Dysfunction	Fischer Exact	0.790
Tile type Fracture	Infection	Logistic Regression	0.090
Tile type Fracture	Chronic pain		0.017
Tile type Fracture	Hannover score		0.500
Tile type Fracture	Majeed score		0.270
Urogenital injury	Sexual dysfunction		0.005
Operation days	Majeed pelvic score		0.2
Operation days	Hannover score		1.0
Polytrauma	Majeed Score		1.0

Legend: Results after 5th paragraph

## Materials And Methods

In this cross sectional study, complex pelvic fracture patients treated by internal fixation in our center during 2011-2014 were collected. The inclusion criteria were patients with open pelvic fracture or unstable pelvic fracture (type B and C Tile classification), which were followed for a minimum of 6 months after internal fixation surgery. Complex pelvic fracture patient with other comorbidities and only have external fixation as definitive treatment were excluded. Complex pelvic fracture cases from 2011 until 2014 were traced from hospital medical record and special pelvic book record. All patient that could be contacted then were being evaluated using the Majeed Pelvic Score and Hannover Score. Several comorbidities, such as chronic pain, sexual dysfunction, and infection had also been assessed. We also recorded several additional data such as gender, type of pelvic fracture, polytrauma status, and history of urogenital injury.

**Fig. 1:**
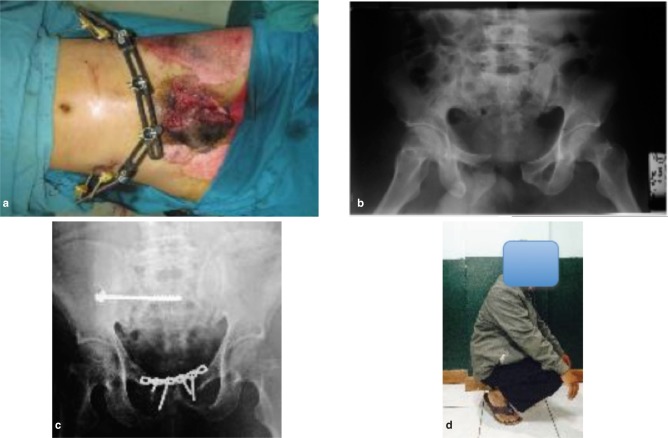
Open Pelvic fracture MTB 1. (a) clinical picture (b) pre operative radiograph (c) post operative radiograph (d) 1 year after operation with excellent majeed pelvic score.

The result was analyzed with SPSS ver.20. All of the association between the variable were analyzed with Fischer exact test. The Multivariat analysis of complex pelvic fracture and comorbidities and functional score were analyzed by using logistic regression. This study was approved by the institutional research ethic committee of our center (FKUI-RSCM).

## Results

Subject Characteristics

Forty-one complex pelvic fracture patients were found from the hospital database during 2011-2014, 26 patients whom fit the inclusion criteria were recruited. Average age was 30.54 ± 10.8 with follow up term average of 25 months. Injury Severity Score (ISS) mean was 27.2. Hospitalization period average was 40 days and the range between hospital admission to surgery was 12 days.

Most subjects were male (65%), in age group of 20-40 (58%), a closed pelvic fracture (73%), and Tile type B fracture (58%). We had nine cases (35%) of complex pelvic fracture combined with polytrauma cases. Most complex pelvic fractures were accompanied with urogenital injury (54%). Sexual dysfunction (38%) was the most frequent complication, followed by chronic pain (35%). There were no non-union observed in our patients

Of the Tile type B and type C, Excellent Majeed Score were found on 78.6% and 50% cases, respectively. Subject also showed Good Hannover Score in 53.3% type B cases and 72.7% type C cases. In term of open or closed fracture, both had an excellent Majeed score of 66.7%.

Statistical Analysis

Tile type fracture (type B or type C) were statistically insignificant with acquired sexual dysfunction (p>0,05), but were significant with the chronic pain (p=0,017). We also found that urogenital injury was associated with sexual dysfunction (p=0,005). Most of the variable (gender, open or closed fracture, Tile type fracture) was statistically insignificant when compared to the functional score (Majeed or Hannover).

**Fig. 2:**
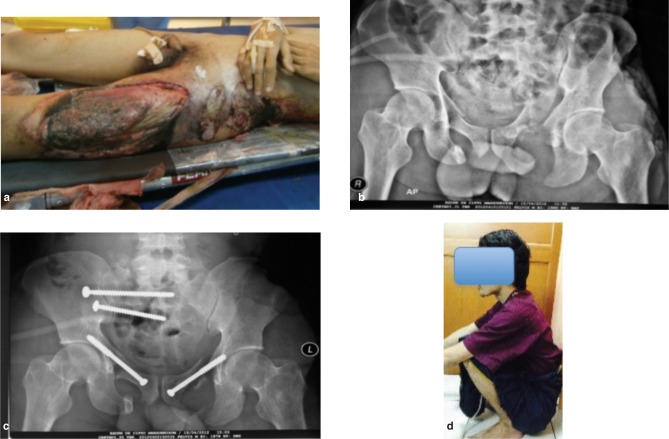
Pelvic fracture MTB 2. (a) clinical picture (b) pre operative radiograph (c) post operative radiograph (d) 2 years after operation with excellent majeed pelvic score.

## Discussion

All complex pelvic fractures cases were resulted from traffic accident. The mean age of patients in this study was 30 years, the age at which patients are active and mobile. This is consistent with the study by Mardanpour *et al*, which the mean age was 37 years^7^. According to the literature, more than 90% of pelvic fractures that occurred is the result of traffic accidents.

Open pelvic fractures have worse functional scores than closed pelvic fractures^8-9^. These difference also occur due to the small number of patients with open pelvic fractures and usually are correlated with other comorbidities^10^. In contrary,

our study didn’t show any statistically signifant difference between the functional outcome of open and closed pelvic fracture. But, open pelvic fracture showed more postoperative infection (42.9%) compared to 10.5% infection in closed series. The closed series has a quite high rate of infection which is probably caused by the inclusion of the closed degloving case in closed fracture group.

Overall the functional outcome after internal fixation in complex pelvic fracture in our center were satisfying, 90 % patients have good and excellent outcome. Our finding slightly exceeds the results from both Pohlemann and Mardanpour study, 81 % of Tile type B cases were excellent and good functional score, while for Tile type C, excellent and good functional score only reached in 73% cases^11-12^. Both research also explained that the percentage of excellent and good functional score in Tile type B fracture is better than Tile type C fracture^13-14^. The higher functional outcome in our series was possibly due to the advance of the minimal invasive technique applied in our series, which was not that common during the time the previous papers were published. There is also no association between polytrauma status and functional outcome of the complex pelvic fracture patients^15^.

Sexual dysfunction is one of the most common complication in complex pelvic fractures^16-17^. This morbidity can reach 61% of complex pelvic fracture cases^18-20^. The rate of sexual dysfunction that occurred in our hospital is lesser compared with other studies. Approximately 21% of patients who experience sexual dysfunction still wear an urocatheter that interferes with sexual activity.

Chronic pain is a frequent complication arised in complex pelvic fracture that degrade the patient’s quality of life^21-22^.

Langford *et al* and Pohlemann *et al*explained that chronic pain often occurs in Tile type C fracture^23-24^. Chronic pain that occurred more in type C fractures was associated with the prevalence of sacroiliac joint pain and leg length inequality in these cases. Meyhoff et al described persistent nerve lesions in 40% of patients with AO C and 5% with AO B fractures contributed to the chronic pain that occurred in their series.

In this study, there were no differences in functional outcome if we perform internal fixation before or after 10 days. Difference with Burkhardt *et al*study^25^, that concluded internal fixation better performed below 7 days to get optimal outcome, is most likely caused by the good preparation and intra-operative management is also essensial for the definitive treatment.
